# Mitral valve repair for infective endocarditis after esophageal reconstruction: a case report

**DOI:** 10.1186/s40792-024-01836-7

**Published:** 2024-02-09

**Authors:** Shingo Tsushima, Yutaka Iba, Tomohiro Nakajima, Junji Nakazawa, Tsuyoshi Shibata, Akihito Ohkawa, Itaru Hosaka, Ayaka Arihara, Nobuyoshi Kawaharada

**Affiliations:** Department of Cardiovascular Surgery, Sapporo Central Hospital, Minami-9, Nishi-10, Chuo-Ward, Sapporo-City, Hokkaido 064-0809 Japan

**Keywords:** Mitral valve repair, Infective endocarditis, Right thoracotomy, Extra-anatomical esophageal reconstruction

## Abstract

**Background:**

In patients with retrosternal neo-esophageal conduit after right thoracotomy, the approach to cardiac surgery could be challenging. Particularly, in patients with infective endocarditis, there is a risk of injury to the conduit through standard median sternotomy. Moreover, right lung adhesions could be predicted. Herein, we present a case of successful mitral valve repair in a patient with infective endocarditis through a redo right thoracotomy after esophageal reconstruction.

**Case presentation:**

A 66-year-old male patient was diagnosed with infective endocarditis and a large anterior mitral leaflet vegetation after a previous esophageal reconstruction via right thoracotomy for esophageal cancer. Due to the retrosternal esophageal reconstruction, we performed a mitral valve repair through a redo right thoracotomy. After resecting the vegetation, the defect was closed with a fresh autologous pericardial patch. Mitral valve annuloplasty was performed. Postoperatively, antibiotics controlled the infection. The patient was discharged on postoperative day 30.

**Conclusions:**

Successful mitral valve repair was performed for infective endocarditis through a redo right thoracotomy after esophageal reconstruction.

## Introduction

Since long-term postoperative outcomes for esophageal cancer improve [1], the case for cardiac surgery after esophageal reconstruction is expected to increase. In patients with retrosternal neo-esophageal conduit after right thoracotomy, determining the approach to cardiac surgery could be challenging. Particularly, in patients with infective endocarditis, there is a risk of injury to the conduit through a standard median sternotomy. Moreover, right lung adhesions could be predicted. We report a case of successful mitral valve (MV) repair of infective endocarditis through a redo right thoracotomy.

## Case report

**Fig. 1:**
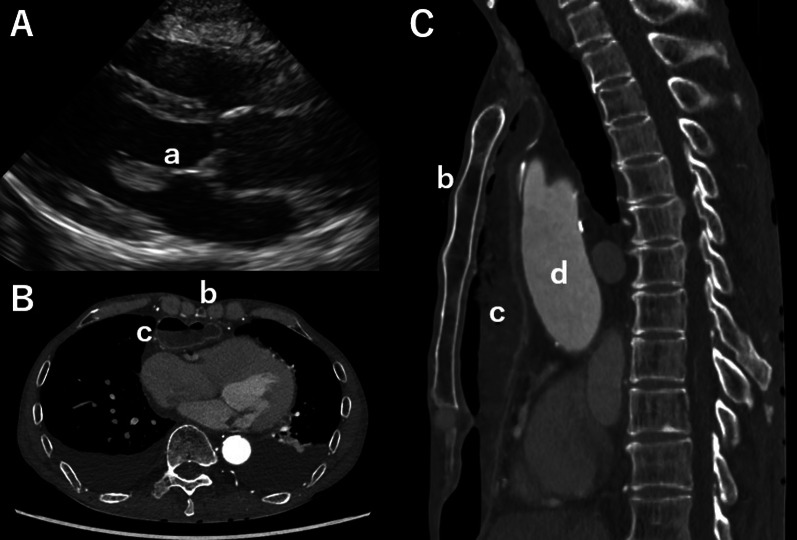
Preoperative transthoracic echocardiography and contrast-enhanced computed tomography findings. **A** Parasternal long-axis view showing a vegetation attached to the anterior leaflet of the mitral valve (a). **B** An axial image of the relationship of the sternum (b) and gastric tube (c). **C** A sagittal image of the conduit and its relationship with the ascending aorta (d)

A 66-year-old male patient who had undergone esophageal reconstruction via the retrosternal route 17 years ago was admitted to the hospital for left hemiparesis. The patient was diagnosed with an acute ischemic stroke, wherein endovascular clot retrieval was performed. The paralysis and disturbance of consciousness promptly improved. However, a fever was noted, and a blood sample cultured *Enterococcus faecalis* growth. Transthoracic echocardiography revealed a large vegetation (24 mm × 9 mm) attached to the anterior leaflet of the MV (Fig. 1A). However, mitral regurgitation was trivial. Contrast-enhanced computed tomography revealed the gastric tube to be located posterior to the sternum (Fig. 1B, C). The distance between the sternum and the vertebrae was 84 mm. To prevent embolism due to the large vegetation, we decided to perform surgery 17 days post-stroke. The surgical strategy was discussed. Median sternotomy posed a risk of injury to the esophageal conduit. Therefore, MV repair was performed through a redo right thoracotomy despite predicting right lung adhesions.

**Fig. 2:**
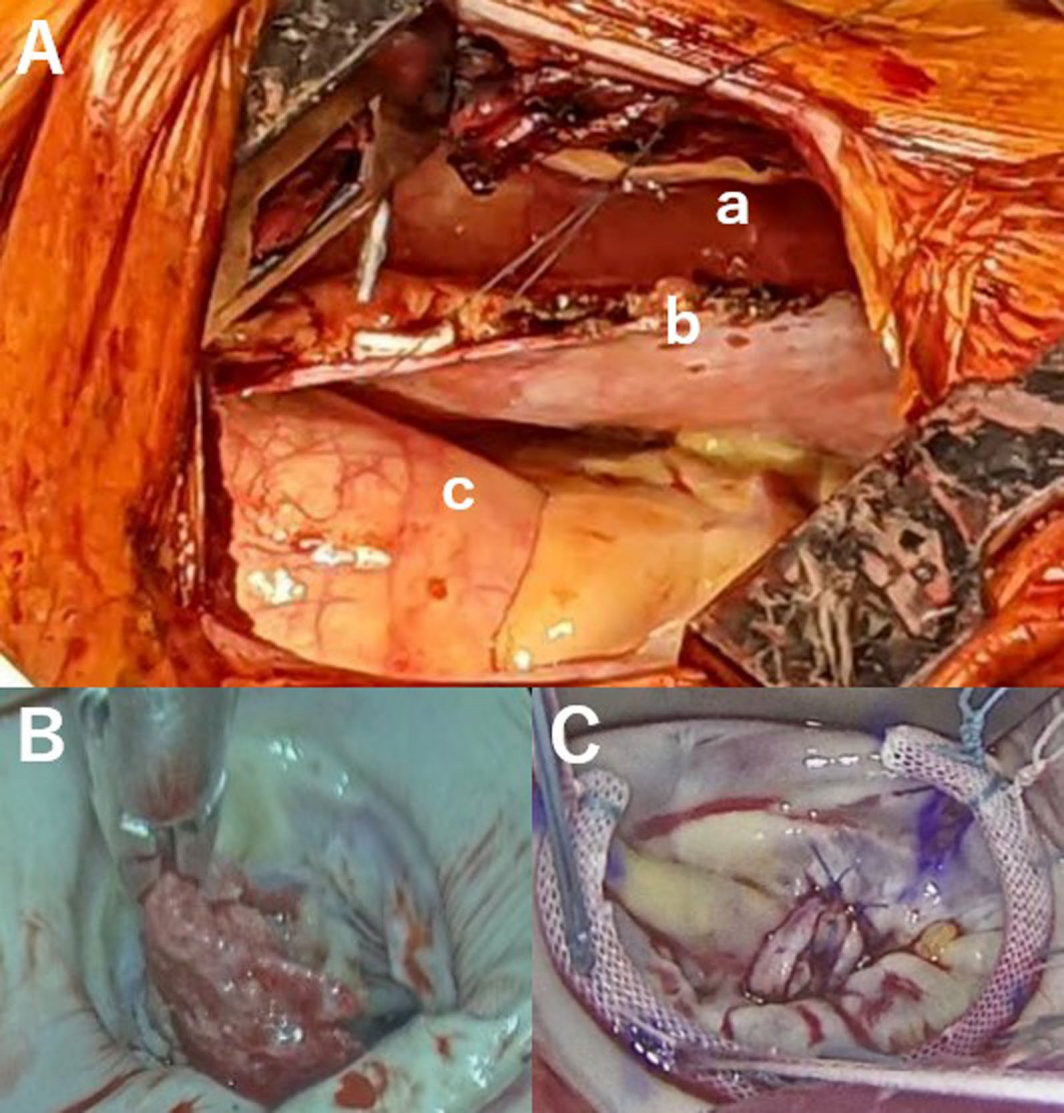
Surgical view through the right thoracotomy. **A** Relationship of the gastric tube (a), pericardium (b), and ascending aorta (c). **B** Vegetation on the anterior mitral leaflet. **C** Mitral valve repair

The patient was intubated using a double-lumen endotracheal tube. The pleural cavity was opened through the right fourth intercostal space with a 10-cm skin incision. Severe adhesions were observed between the right lung and chest wall. After dissection of the right lung adhesions under direct vision, the pericardium was opened anterior to the phrenic nerve and lifted to the chest wall to avoid damaging the reconstructed gastric tube (Fig. 2A). Five pericardial retraction sutures were used for elevating the heart. Cardiopulmonary bypass was established using right femoral artery cannulation and right jugular and right femoral vein drainage. The ascending aorta was cross-clamped using a flexible aortic clamp. In addition, antegrade cardioplegia was administered. The MV was visualized through a right-sided left atriotomy (Fig. 2B). The distance between the sternum and the vertebrae was a little short. However, the pericardium was lifted to the chest wall to cover the gastric tube, and the MV visualization was generally acceptable. After resecting the vegetation from the A2 portion of the anterior mitral leaflet, the defect was closed with a fresh autologous pericardial patch using seven pairs of mattress sutures. Mitral valve annuloplasty (Fig. 2C) was performed using a 30-mm Physio Flex Annuloplasty Ring (Edwards Lifesciences, Irvine, CA, USA). The aortic cross-clamp was removed after closing the left atriotomy. Cardiopulmonary bypass and aortic cross-clamp durations were 193 and 140 min, respectively.

Postoperatively, the infection was controlled using antibiotic therapy with ampicillin for 4 weeks and gentamicin for 2 weeks. Postoperative transthoracic echocardiography revealed trivial mitral regurgitation and no sign of recurrent infection. No postoperative neurological events were observed. The patient was discharged on postoperative day 30.

## Discussion

In recent years, the retro-sternal route has tended to be preferred over the posterior mediastinal route for esophageal reconstruction in Japan [2]. Therefore, it is possible that cases similar to our case will increase. The optimal approach of surgery in a patient with a retrosternal neo-esophageal conduit after a right thoracotomy could be challenging to determine. Particularly in cases requiring complicated procedures, such in those with infective endocarditis, owing to the risk of injury to the conduit during standard median sternotomy and presence of right lung adhesions, determining the approach to cardiac surgery could be difficult. Inra et al. have performed median sternotomy in four patients after extra-anatomical esophageal reconstruction with a retrosternal or subcutaneous neo-esophagus: two and other two patients underwent left thoracotomy and right thoracotomy, respectively [3]. Delayed injury to the retrosternal conduit after median sternotomy occurred in one patient. We suggest that the thoracotomy approach could help avoid conduit injury. Wakasa et al. have described performing aortic valve replacement through a left thoracotomy after esophageal surgery with substernal gastric tube reconstruction [4]. Furthermore, Numaguchi and Shiiku have performed MV replacement and coronary artery bypass grafting through a left thoracotomy after retrosternal esophageal reconstruction [5]. Left thoracotomy was considered unsuitable for MV repair, as evaluating the repaired MV during displacement of the heart was difficult. We performed MV repair through a redo right thoracotomy and evaluated the quality of the repair using the saline test.

Prestipino et al. have suggested that using less-invasive techniques during redo surgery could minimize morbidity and mortality without prolonging the duration of cardiopulmonary bypass [6]. There are three types of minimally invasive cardiac surgery (MICS) via right thoracotomy: direct, thoracoscopic, and robotic [7]. Direct MICS, which we performed, could create a larger wound compared with other procedures. However, the dissection area between the lung and chest wall could be minimized because of manipulation in a single window. If ports are inserted, as in endoscopic or robotic MICS, the dissection area becomes wider. In our case, the adhesion was severe, and there was mild lung injury. However, air leakages could be repaired using fibrin glue and a polyglycolic acid sheet. There was no air leak from the thoracic drain postoperatively.

In leaflet manipulation associated with infective endocarditis, autologous pericardium is often required [8]. Although the anterior mediastinum was manipulated during esophageal reconstruction, the right lateral side of the pericardium had less inflammation than did the anterior one; consequently, it was easily harvested during right thoracotomy and could be used for leaflet repair.

## Conclusion

We encountered a case of successful MV repair in a patient with infective endocarditis treated with redo right thoracotomy after retrosternal esophageal reconstruction.

## Data Availability

Data underlying this article will be shared on reasonable request to the corresponding author.

## Abbreviations

MV: Mitral valve
MICS: Minimally invasive cardiac surgery
